# A hybrid machine learning model of depression estimation in home-based older adults: a 7-year follow-up study

**DOI:** 10.1186/s12888-022-04439-4

**Published:** 2022-12-21

**Authors:** Shaowu Lin, Yafei Wu, Ya Fang

**Affiliations:** 1 The State Key Laboratory of Molecular Vaccine and Molecular Diagnostics, School of Public Health, Xiamen University, Xiamen, 361102 China; 2 National Institute for Data Science in Health and Medicine, Xiamen University, Xiamen, 361102 China; 3 Key Laboratory of Health Technology Assessment of Fujian Province, School of Public Health, Xiamen University, Xiamen, 361102 China

**Keywords:** Machine learning, LSTM, Depression, Home-based elderly, Prediction

## Abstract

**Background:**

Our aim was to explore whether a two-step hybrid machine learning model has the potential to discover the onset of depression in home-based older adults.

**Methods:**

Depression data (collected in the year 2011, 2013, 2015 and 2018) of home-based older Chinese (*n* = 2,548) recruited in the China Health and Retirement Longitudinal Study were included in the current analysis. The long short-term memory network (LSTM) was applied to identify the risk factors of participants in 2015 utilizing the first 2 waves of data. Based on the identified predictors, three ML classification algorithms (i.e., gradient boosting decision tree, support vector machine and random forest) were evaluated with a 10-fold cross-validation procedure and a metric of the area under the receiver operating characteristic curve (AUROC) to estimate the depressive outcome.

**Results:**

Time-varying predictors of the depression were successfully identified by LSTM (mean squared error =0.8). The mean AUCs of the three predictive models had a range from 0.703 to 0.749. Among the prediction variables, self-reported health status, cognition, sleep time, self-reported memory and ADL (activities of daily living) disorder were the top five important variables.

**Conclusions:**

A two-step hybrid model based on “LSTM+ML” framework can be robust in predicting depression over a 5-year period with easily accessible sociodemographic and health information.

**Supplementary Information:**

The online version contains supplementary material available at 10.1186/s12888-022-04439-4.

## Introduction

Depression is the most common psychological problem in older people. Almost 7% of older adults worldwide are currently suffering from depressive disorder [1]. Globally, the COVID-19 pandemic has added a 27.6% increase of depression [2]. Geriatric depression usually impairs an individual’s life style and even physical functioning, and could severely affect the work and daily life of older adults [3]. In China, the prevalence of 11.5% to 21.1% in depression gave a more sever challenge [4]. Thus, the need of estimating depression earlier and accurately is more and more urgent.

However, depression forecasting is considerablely difficult and dominated by various risk factors. Several attempts have been made to predict depression utilizing regression models such as generalized linear regression, and machine learning techniques with demographic characteristics, social factors and health data. For instance, Tsai Y F et al. compared the severity of depression and its main influencing factors among nursing home elderly in different regions, in which a total of 214 older adults from Hong Kong and 150 older adults from Taiwan were included. Logistic regression analysis suggested that life satisfaction, gender, income, and self-reported health had a great impact on depression prediction of nursing home elders. While for Hong Kong elders, significant predictors of depression were functional status, cognition, and life satisfaction [5]. Furthermore, Chang et al. used the multinomial logistic regression to explore the depression development trajectory of the elderly in Taiwan by gender stratification, and the results found that old aged men with less social support had a higher burden of depression. However, due to the limitations of traditional regression methods in handling high-dimensional and non-linear data, their performance is often limited [6]. Also, compared with traditional regression approaches, machine learning-based models have a superior predictive ability [7]. Several studies using ML algorithms (e.g., RF) have found significant improvements in the accuracy of predicting depression [8, 9]. Helen et al. [10] used decision tree to uncover non-linear associations and interactions among physical and mental health factors, as well as cognition and magnetic resonance imaging in depressed older adults through investigating a total of 81 participants (51 major depressive disorder patients, 30 controls). They found that executive function and cognitive tests of verbal fluency had the best influencing prediction for late-life depression. Also, the results further suggested the direct association between depression and cognition. Aris Supriyanto [11] used C4.5 algorithm to estimate the risk of postpartum depression, and they found the greatest profit on the blood pressure, psychological variables, body temperature, indicating the greater impact of the three factors on the depressed individuals, and should be prioritized for intervention and treatment. Md. Rafiqul Islam et al. [12] performed depression analysis on 7145 Facebook data obtained from an Australia online social media platform. To investigate identification of depression, they proposed three machine learning techniques, namely decision trees, k-nearest-neighbor, support vector machine. The results showed that the predictive performance (accuracy) of support vector machine was superior to other ML approaches (99%, 96%, 88%). In addition to the above commonly used methods, association analysis, frequent pattern trees, artificial neural networks, and ensemble learning (such as GBDT: gradient boosting decision tree) have also been used in depression risk research [13]. For example, Thanathamathee et al. [14] compared adolescent depression with a consideration of severity (light, medium, and severe), the study found that AdaBoost algorithm significantly improved the performance of constructing model (accuracy: 82.7%). Additionally, in order to explore the physiological characteristics of depressed patients in depth, Yusra Ghafoor et al. [9] applied frequent pattern tree and association analysis on depression database containing 5,964 records and found that sleep disorders, sleepless nights or sleep too much, easily fatigue are the most important clinical symptom of depressed patients. Although the above classifiers were powerful in prediction tasks, their performance was still limited due to the lack of capturing temporal information in the traditional features. Thus, a modern deep learning algorithm of long short-term memory network (LSTM) was proposed to capture the time-sensitive features, which could be conveniently used to predict the future stage of the disease. For the purpose of improving the prediction of depression in the long-term period, Xu et al. developed a deep neural network framework (LSTM) for the individualized prediction of depressive disorder based on 22-year longitudinal community dwelling data among older adults in the United States (AUC=0.87) [15].

Depression is marked by a chronic and longer-term course. To date, numerous studies have been published using longitudinal cluster analysis to examine the course of depression and examine heterogeneity in trajectories of depression. Some studies also have investigated the determinants of its heterogenous course. Indeed, a temporal relation can be found among these factors. Studies on the determinants of depression have shown that such risk factors may evolve over time. Yet, previous studies on the association between depression and predictors largely dominated by features at a single wave that usually cannot take time-varying features into account, and thus they may not fully reflect how dynamic and stability of these features have an affect on depression. Thus, exploring the time-dependent relationship between these risk factors can help medical workers better predict the risk of depression. Moreover, studies estimating depression for population of home-based older adults are scarce, but home-based eldercare will remain the main selection for older Chinese in a long-term period of future, due to the ethics of Chinese “filial piety” [16].

To the best of our knowledge, few studies have focused on the prediction of depression among home-based older adults in the next few years using longitudinal data. In the current study, we aim to propose a two-step hybrid machine learning model for depression classification and investigate the following: 1) are the LSTM algorithm a suitable model to predict the level of different depression risk factors in home-based older adults in the next two years by capturing time series informations; 2) how do three ML classifiers compare to classical regression model in terms of predicting the onset of depression in the home-based elderly population in two years; 3) which features are important predictors of depression for home-based older adults in a large Chinese community cohort based on the material of demographic, social economy, lifestyle and health status, and can new features be recognized in addition to established risk factors?

## Methods

### Study design and participants

**Fig. 1:**
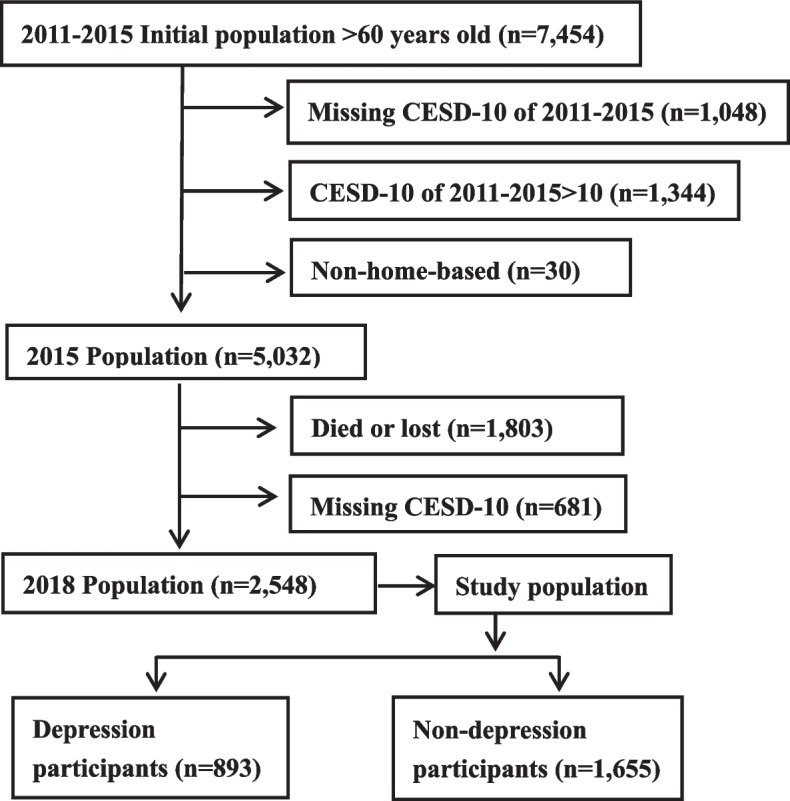
A flow chart for study population selection. CESD-10:10-item Center for Epidemiologic Studies Depression Scale

This study was a secondary analysis of data obtained in the China Health and Retirement Longitudinal Study (CHARLS). Specific details of this nationwide study have been reported in previous studies [17]. The protocol had received permission from the Biomedical Ethics Committee of Peking University, and an institutionally informed consent form was signed by all participants. 2548 home-based older adults (60+ years) with community dwelling data obtained in the year of 2011, 2013, 2015 and 2018 were used in the current study, in which participants with a CESD-score >10 in wave 1, 2 and 3 were excluded (Fig. 1). A summary of missing values of all predictors were listed in Supplementary Table 1. The missing values were replaced utilizing the multiple imputation method, which is based on 5 duplicates and a chained equation, using the R “mi” package. Besides, a sensitivity analysis was conducted only on participants with complete data to evaluate the robustness of our models.

### Predictor variables

In our study, 22 variables were included as candidate predictors. Specifically, 24 predictors firstly derived from the previous forecasting study of depression in literature [18–20], especially machine learning (ML) research [19, 21], were selected as prioritized. Secondly, we selected the same predictors as in the wave 1-3 due to the difference in the variable structure of the questionnaires under different waves and thus the variable of major misfortune injury experience was removed. Similarly, the variable of CESD-10 score was also not included as a categorical predictor since the participants with a CESD-score >10 in wave 1, 2 were excluded. Thirdly, we reduced the number of candidate predictors to a proposed event per variable (EPV) value of 10 (i.e., 10 cases per predictor) [22] to overcome methodological limitation, such as selecting unimportant variables. Finally, a set of 22 variables (divided into three groups) in the year 2011 were selected as predictors (Supplementary Table 4). Details of these variables are presented as follows:

- For demographic variables, geographical location (eastern vs. central vs. western) [23], age [24], sex [20], rural/urban community [25], marital status [26] were included, and age and sex were regarded as auxiliary input because they are variables that are not needed to predict over time. Geographical location was divided into eastern, central, and western regions according to the 2011 China health statistics yearbook. Marital status was categorized as married (married/partnered), and single (never married/divorced/separated and widowed).
- For socioeconomic variables, we considered educational level [27], household per capita income [28], household registration [29], occupational status [30], medical insurance (yes/ no) [31]. Education level was dichotomized as low-level (elementary school and below) and high-level (middle school and above). Household per capita income was defined as total household income divided by the number of people living in the family, and was grouped into three categories based on an interval of 5000 RMB. Household registration was categorized as agriculture, non-agriculture and not registered. Occupational status was divided into agricultural work, non-agricultural work, retired and unemployed/never work [23].
- For variables in lifestyle and health status, cognitive ability [32], sleeping time [33], self-rated memory [34], life satisfaction [35], ADL (activities of daily living) disorder [36], self-reported health status [37], social activities in the past month [38], smoking [39], alcohol drinking [27], chronic diseases [40], disability [41], medical services experience in the past month [42] were selected as predictors. Consistent with previous studies [43], cognitive ability was evaluated by episodic memory and mental intactness, and the global cognitive scores were calculated as the sum of the scores of episodic memory and mental intactness with a range from 0 to 21 [44]. In order to better observe the cognitive ability, the total cognition score was divided into two categories: above-average cognitive scores (high: score>10.5) and under-average cognitive scores (low: score<10.5) [45]. ADL impairment was measured by asking participants whether they had any difficulties in taking a bath, eating, getting in and out of bed, dressing, using the toilet, defecating, doing housework, cooking, making phone calls, taking medicine, shopping and managing finances due to health and memory problems in the past 3 months, which was previously reported by Katz [46] and Lawton [47]. If any difficulty was reported, the participant would be classified as having difficulties in ADL. Social activities experience (over the past month) covers interaction with friends, voluntary or charity work, stock investment, and other 8 kinds of social activity. Participants who had been diagnosed with hypertension, dyslipidemia, diabetes or high blood glucose, cancer, chronic lung diseases, and other 9 kinds of chronic disease were defined as having chronic disease. Medical services experience refers to a participant who have visited a hospital, or doctor’s practice, or been visited by a doctor for outpatient care in the last month. Major misfortune injury experience refers to a participant who had ever been injured in a traffic accident or any other major accidents.

### Outcome variables

The 10-item Center for Epidemiologic Studies Depression Scale (CESD-10, Supplementary Table 5) was applied for assessing depression symptoms during the 4 waves of survey. The reliability and validity of CESD-10 has been validated in Chinese older population, Cronbach's alpha = 0.86 [48]. Respondents were asked about the number of days they experienced with different emotions during the past week, and a respondent who had a CESD-10 score of at least 11 was defined as suffering depression in our study [49].

### Data analysis

**Fig. 2:**
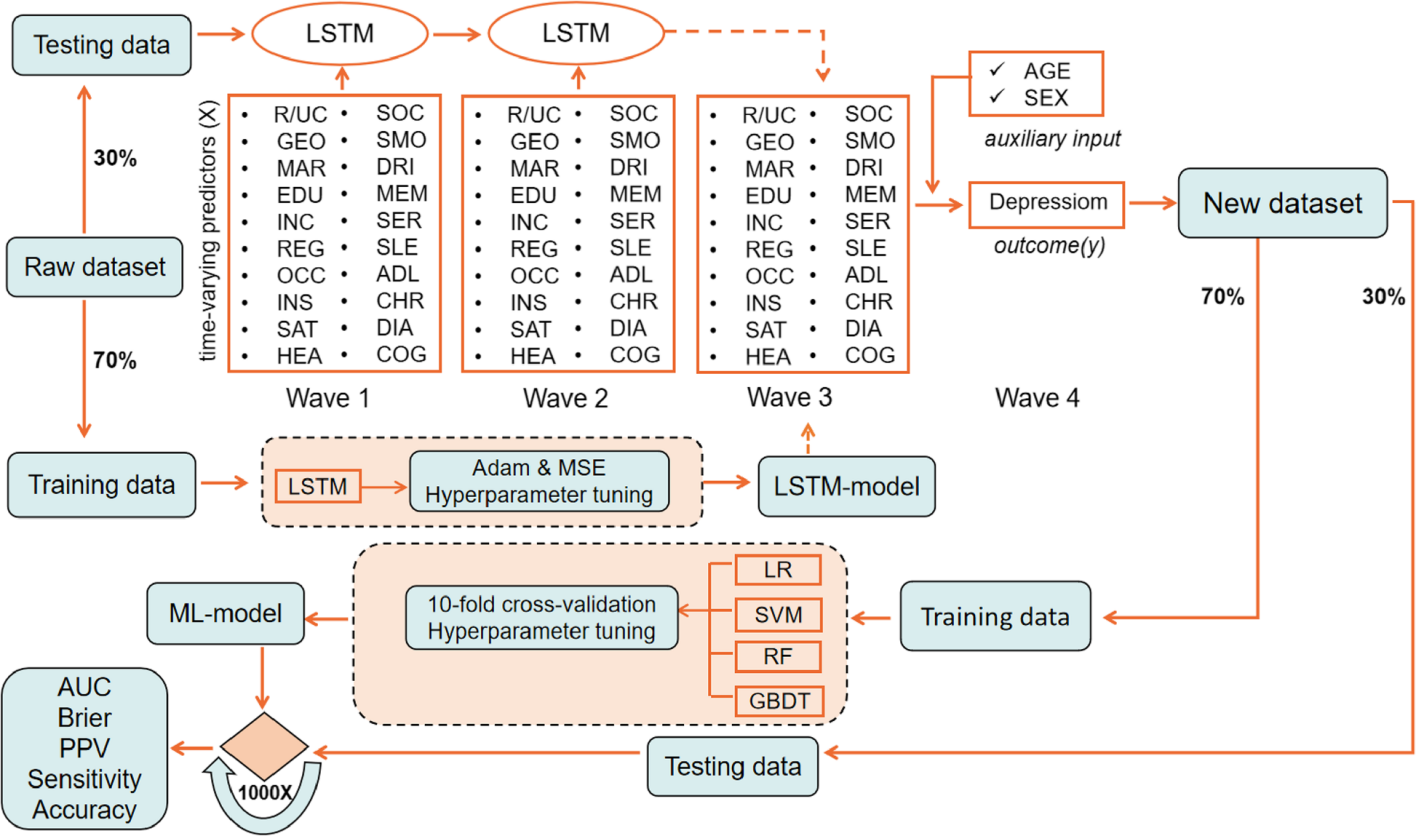
The architecture of the hybrid model for estimation of depression. Raw dataset used Wave 1-3 of depression data, and the output predicted data of Wave 3 and the outcome of wave 4 were constructed into a new dataset. MSE: mean squared error; ML: machine learning; RF: random forest; GBDT: gradient boosting decision tree; SVM: support vector machines; LR; logistic regression. R/UC: rural/urban community, GEO: geographical location, MAR: marital status, EDU: education level, INC: household per capita income, REG: household registration, OCC: occupation status, INS: medical insurance, SAT: life satisfaction, HEA: self-reported health status, SOC: social activities, SMO: smoking, DRI: drinking, MEM: self-rated memory, SER: medical service, SLE: sleeping time, ADL: ADL disorder, CHR: Chronic disease, DIA: Disability, COG: Cognitive ability

The depression prediction task mainly consisted of two steps (Fig. 2). In the first stage, we performed the LSTM algorithm to estimate the value of risk factors of participants in 2015 utilizing the first 3 waves of data, which constituted a raw dataset. More specially, we used the categorical predictors in waves 1-2 to predict the corresponding factors of depression for elderly in wave 3 (2015) using a randomly data separation method, that is, 70% of the samples for training the LSTM model, and the remaining 30% of the samples, a validation dataset, was used for prediction to ouput the predicted factors in 2015. In the second stage, three commonly used machine learning (ML) approaches were applied to investigate whether the predicted features of 2015 could accurately predict depression in wave 4 (2018), and the output factors from the validation dataset were combined with the depression results from wave 4 to form a new dataset, in which 70% of the data was used for model construction, and the remaining 30% of data was used for model testing. Model performance was evaluated by metrics of accuracy, sensitivity, positive predictive value (PPV) and AUROC. In addition, brier score was selected as the calibration index. The construction and evaluation of machine learning methods are completed by python 3.7 with Pytorch and Scikit-learn toolkit. A bilateral *p* value of <0.05 was considered statistically significant. The feature importance was explored by calculating Shapley values via the SHAP package (v. 0.39.0) and visualized by beeswarm plot.

Five prediction algorithms were employed in our study: the binary logistic regression model (LR), a typical conventional statistical model, and three commonly ML models, including random forests (RF), gradient boosting decision tree (GBDT), and support vector machines (SVM); and a time-series based deep learning method (long short-term memory network: LSTM). SVM is a controlled classification algorithm based on statistical learning theory. The core of SVM is based on the concept of finding the most appropriate decision function that separates the two distinct classes on the basis of the definition of hyperplane, which can distinguish the two classes from each other in a most appropriate way [50, 51]. GBDT is a flexible and non parametric statistical learning technique for classification and regression. It improves the prediction appearance results by gradually improving the estimation [52]. RF is an ensemble algorithm that inputs the same test dataset to all learned decision trees and collected the results by the majority vote method. It has high prediction power for high-order data, which have a large number of explanatory variables and complicated interactions between them [53]. Since the above models are traditional statistical and machine learning models, both of which are shallow learning models, and cannot capture the interdependence of predictors in different bands of longitudinal data during the prediction process. Therefore, we proposed LSTM algorithm, a special kind of recurrent neural network (RNN), to capture the interdependence of predictors in different waves of longitudinal data and estimate the future trend of predictors. LSTM, with the chain of repeating neural network modules, is designed to avoid the long-term dependency problem, which might be able to connect previous information to the present task and encode the temporal relation between features [54]. The adjustment of the hyperparameters is essential for model construction. For LSTM model, the learning rate is one of the most important parameters that can directly affect the convergence of the model. We repeatedly tried the value of learning rate range from 1e-1 to 1e-4 during the training process. Adam optimizer and MSE (mean squared error) of loss function were used. We evaluated the training performance of LSTM by looking at the training curve, and a steadily decreasing loss value was observed in Supplementary Figure 1. Considering that the fitting result of the training set would affect the generalization ability of the test set, once the test error stopped falling or the error started to increase, we have to stop training. The hyperparameter settings are shown in Supplementary Table 6. A standard machine learning technique is trained on the training set, and then were evaluated on the test set. For each analysis, we randomly split the whole data into training (70% of the entire sample) and testing datasets (30%) [55]. We deployed a standard machine learning protocol with 10-fold cross-validation [56], hyperparameter tuning, using the training dataset. The detailed information about hyperparameter tuning strategy of three ML algorithms was listed in Supplementary Table 7. In the model evaluation, the accuracy, sensitivity, PPV (positive predictive value), and AUROC of the discrimination metrics were selected to evaluate the prediction performance of the proposed models. In addition, brier score was used to reflect the calibration of models and all the testing process was repeated 1000 times to take the average of those outputs as a stable estimation (Fig. 2). For the RF and GBDT classifiers, we further calculated the relative importance of the predictors according to their contribution to prediction accuracy.

## Results

Table 1 summarizes the 20 predicted features and 2 auxiliary predictors of 2015 for depressed and no-depressed participants in wave 4 (year of 2018) using the validation dataset. The proportion of males for elderly in no-depressed and depressed group were 56.8% and 52.6%, respectively. There were significant differences between two groups by rural/urban Community, household per capita income, self-reported health status, drinking, disability, cognitive ability. The baseline characteristics was presented in Supplementary Table 2.

**Table 1:** Predicted characteristics in 2015 and univariate analysis of association with depression in 2018 based on the validation dataset

Variables	Non-depression (*n*=532)	Depression (*n*=232)	Test of association
	% or Mean (SD)	% or Mean (SD)	
Demographic variables			
Age, year			
60-	32.36	35.26	χ^2^=0.51
70-	47.40	42.51	
80-	20.24	22.23	
Sex			
Male	56.77	52.59	χ^2^=1.14
Female	43.23	47.41	
Rural/Urban Community			
Rural	58.83	69.40	χ^2^=7.65**
Urban	41.17	30.60	
Geographical location			
Eastern	35.90	39.66	χ^2^=1.91
Central	56.58	51.29	
Western	7.52	9.05	
Marital status			
Single	22.74	25.86	χ^2^=0.869
Married	77.26	74.14	
Social economy variables			
Education level			
Low	91.73	92.67	χ^2^=0.196
High	8.27	7.33	
Household per capita income, yuan			
<5000	0.38	2.59	χ^2^=7.62**
5000-10000	99.62	97.41	
Household registration			
Agriculture	85.15	86.21	χ^2^=0.95
Non-Agriculture	14.85	13.79	
Occupation status			
Agricultural work	6.02	8.19	χ^2^=1.78
Non-agricultural work	77.26	77.59	
Retired	16.73	14.22	
Medical Insurance			
No	7.33	11.21	χ^2^=3.12
Yes	92.67	88.79	
Life style & health status variables			
Life satisfaction			
Satisfied	9.40	8.62	χ^2^=0.121
Medium	57.89	58.62	
Not satisfied	32.71	32.76	
Self-reported health status			
Good	25.38	9.91	χ^2^=92.95***
Fair	55.83	37.50	
Poor	18.80	52.59	
Social activities			
Never	54.51	51.29	χ^2^=0.67
Ever	45.49	48.71	
Smoking			
Never	73.50	71.12	χ^2^=0.46
Ever	26.50	28.88	
Drinking			
Never	63.53	77.16	χ^2^=13.71***
Ever	36.47	22.84	
Self-rated memory			
Good	32.71	29.31	χ^2^=0.96
Fair	51.32	53.02	
Poor	15.98	17.67	
Medical service			
No	81.95	80.17	χ^2^=0.34
Yes	18.05	19.83	
Sleeping time, hour			
0-	15.60	13.79	χ^2^=0.699
4-	25.94	26.29	
6-	27.44	29.74	
8-	31.02	30.17	
ADL disorder			
No	73.87	73.28	χ^2^=0.03
Yes	26.13	26.72	
Chronic disease			
No	51.50	55.17	χ^2^=0.87
Yes	48.50	44.83	
Disability			
No	90.23	80.60	χ^2^=13.50***
Yes	9.77	19.40	
Cognitive ability, score			
Low	54.89	71.12	F=17.712***
High	45.11	28.88	

χ^2^ denotes the Pearson’s Chi square statistic; *p < 0.05, **p < 0.01, ***p < 0.001

Supplementary Figure 2 presents the correlation of each predicted risk factors by LSTM model. We found their correlation coefficients of all pair-wise features were lower than the value of 0.7, and the largest relation was observed between cognitive ability and chronic disease, achieved 0.64.

**Fig. 3:**
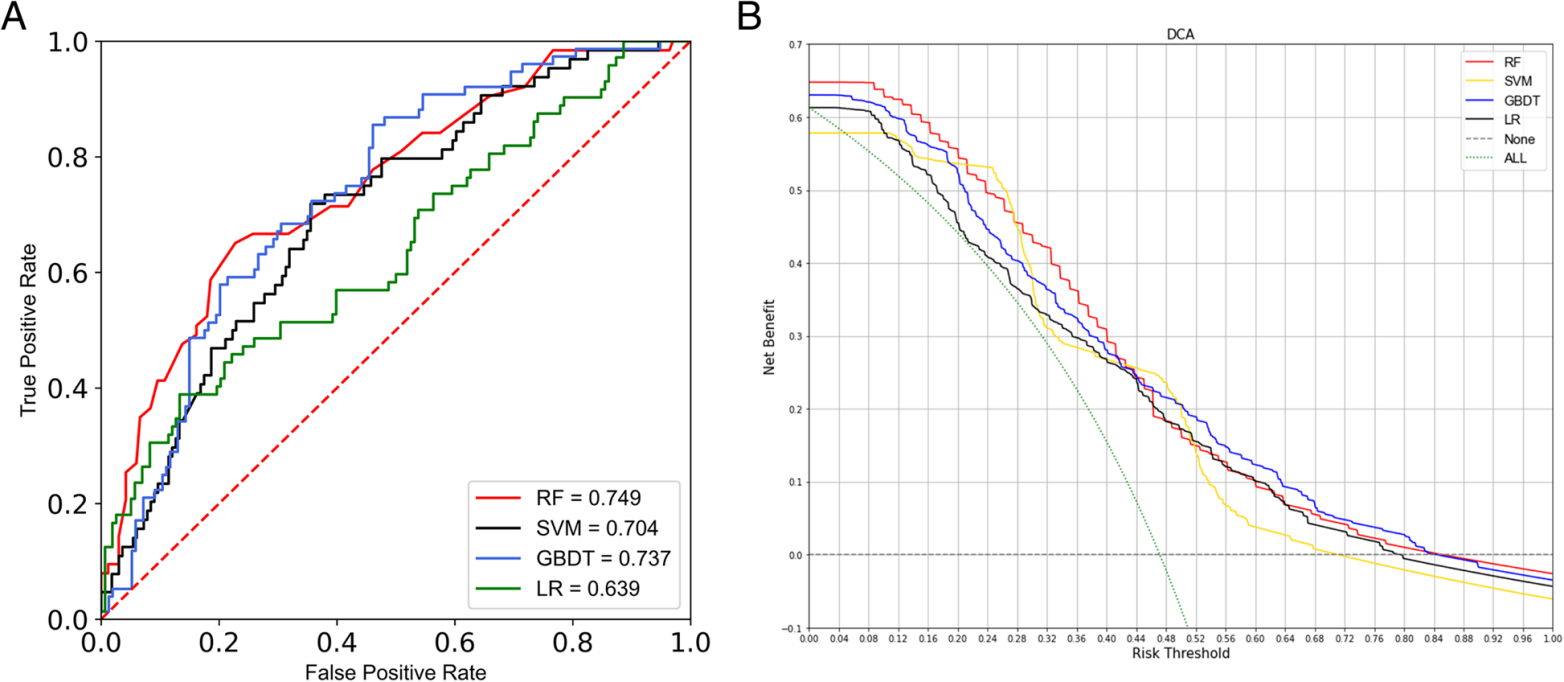
Predictive performance of three ML and LR for estimation of depression (A, ROC curve. The x-axis represents specificity (probability of negative test given that the elderly did not have the depression), and the y-axis represents sensitivity (probability of a positive test given that the elderly had the depression). B, decision curve analysis. The x-axis represents the threshold probability of the depression. The y-axis represents net benefit.). RF: random forest; GBDT: gradient boosting decision tree; SVM: support vector machines; LR; logistic regression

The forecasting performance of LSTM model is shown in Supplementary Figure 1. The mean squared error (MSE) was quite similar between the training and validation curves, both achieved around 0.8, which suggested that the time-series based model had a good fit on the risk factors forecasting in the first task Fig. 3. A shows the ROC curves of different ML models for depression prediction. Generally, RF had the best performance, with an AUC value of 0.749, and the LR was significantly lower than those of other three ML models. RF also had the highest value of PPV (0.650). Although the PPV of SVM model (0.462) was slightly lower, the sensitivity was the highest (0.469) compared with GBDT (0.303), RF (0.206), and LR (0.264). The accuracy of RF, LR, SVM, GBDT were 0.752, 0.713, 0.701, 0.682 respectively. For the calibration metric, the scaled brier score of RF was the lowest, while the values of the other three algorithms were relatively higher, ranging from 0.183 and 0.205 (Table 2). Supplementary Table 3 also suggested the robustness of our models when a sensitivity analysis was performed only on participants with complete data. DCA results (Fig. 3. B) showed that within the threshold ranges of 0.00-0.20 and 0.28-0.40, the net benefit of RF was the largest. The SVM achieved the best net benefit within the threshold ranges of approximately 0.20-0.28 and 0.44-0.48, and within the threshold ranges of around 0.52-0.84, the net benefit of GBDT was much higher.

**Table 2:** Model performance in predicting onset of depression

Model	Accuracy	Sensitivity	PPV	Brier score	AUC (95% CI)
SVM	0.701	0.469	0.462	0.183	0.703 (0.702-0.705)
GBDT	0.682	0.303	0.535	0.198	0.737 (0.731-0.738)
RF	0.752	0.206	0.650	0.170	0.749 (0.744-0.749)
LR	0.713	0.264	0.594	0.205	0.639 (0.633-0.640)

*RF* random forests, *GBDT* gradient boosting decision tree, *SVM* support vector machines, *LR* logistic regression, *PPV* Positive Predictive Value, *CI* confidence interval; Parameter optimization: RF (ntree=200, mtry=40), SVM (C=0.1, γ=0.0001), GBDT (α= 8, β=0.001, γ= 300).

**Fig. 4:**
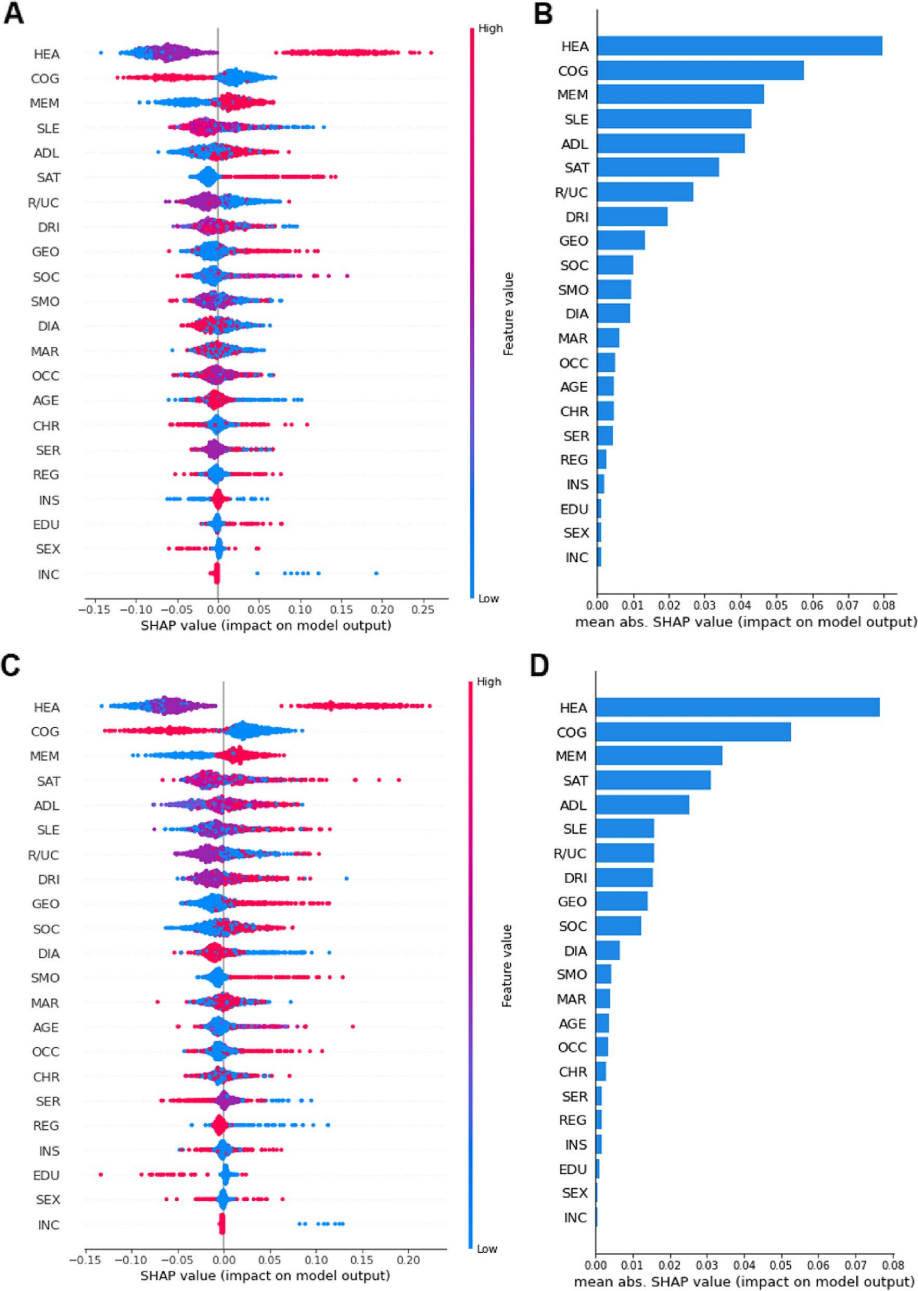
Variables selected by RF and GBDT with relative relevance weights. (A/C) SHAP (SHapley Additive exPlanation) values are ordered by value of a feature to the predictions made by the RF/GBDT. The position on the x-axis on shows whether the effect of that value is associated with a higher or lower prediction for a given observation. Red color indicates the feature is high for that observation or low (blue). (B/D) Summary of mean SHAP values or overall magnitude of a feature’s impact on prediction of depression by the RF/GBDT

Cognitive ability and self-reported health status were consistently ranked as 1st or 2nd important predictors for RF and GBDT models (Fig. 4). The two classification models ranked self-reported memory and ADL disorder as two of the top five important predictors, which was mainly composed of lifestyle and health status variables. While, life satisfication and sleep time were just ranked one of five most important predictors for GBDT and RF, respectively. For demographic variables, geographical location and rural/urban community was two of the top ten important predictors in forecasting the depressed elderly with an exception. Regarding socioeconomic variables, two models ranked occupational status as moderately important predictors (Top 15). However, household per capita income, educational level, medical insurance were consistently the least important predictors in GBDT and RF models.

## Discussion

This study retrospectively collected 4 Waves of CHARLS longitudinal data, a total of 2,548 older adults, in which demographic information, socioeconomic, lifestyle variables and health status were obtained. The results showed that LSTM model could successfully predict the risk factors in the next one year. We also explored the difference of predicted features in two outcomes and the correlation of each predicted features. The founding suggested that there was a weak collinearity between these predictors. RF achieved the best performance for the prediction of depression based on the features predicted by LSTM, and our results revealed that cognitive ability, self-reported health status, self-reported memory, and ADL disorder were top 5 predictors.

In our designed predictive models, we observed the AUCs ranged from 0.639 to 0.749, and accuracies ranged from 0.682 to 0.752. Meanwhile, the mean AUCs of the three ML models were higher than that of traditional statistical model (LR) in the estimation of elderly depression symptoms. Richard Dinga et al. [57] assessed 804 individuals from the Netherlands Study of Depression and Anxiety (NESDA), demonstrated a model with C statistics of 0.66 to distinguish patients with and without a unipolar depression diagnosis at 2-year follow-up. K.-S. Na, et al. examined baseline (2016) and follow-up (2017–2018) data of the Korea Welfare Panel Study (KoWePS) to predict the future onset of depression, achieving AUCs of 0.870. When comparing our proposed models with other similar studies predicting depression on the individual level, our results fall into the upper level of the AUC range (0.65–0.84) [58]. However, only several commonly ML algorithms were used to build predictive model in previous studies, while our proposed hybrid ML framework was scarce in the community-based longitudinal studies.

To detect features importance, the two optimal tree-based models, RF and GBDT, were used to show the interpretability of the inner mechanisms in models’ decisions. Overall, cognitive ability, self-reported health status, self-rated memory, and ADL disorder were the top 5 important predictors. The association between cognitive impairment and depression is still debatable. Two studies reporting no effect of cognitive impairment on depressive population [59, 60] while two other studies reported that cognitive impairment increased risk of developing into depression [37, 61]. ADL disorder was a robust predictor of depression across a number of studies [38]. With the increase of age, older people may be confronted with more health problems that can affect their life independency. Our results provided supportive evidence that older adults having limitations in ADLs were more likely to report depression. In the lifestyle and health status variables, self-reported health status and self-rated memory were also the considerable important predictors, which is similar to the results of Kuo et al. and Liang et al. They found older adults with poor self-rated health were more likely to experience higher symptom burdens [40, 60] . All in all, the higher ranking features in the two models (e.g., cognitive ability, sleeping time, self-reported memory) could share common underlying biological mechanisms, that is, the relationship between the activation of microglia [62] and the onset of depression.

However, neither the marital status nor the chronic diseases [63] were identified as important factors in the current study. Possible explanations might be the heterogeneity of samples, we only divided the marital status into two classification and used the number of chronic diseases instead of the specific types of disease. Therefore, further studies focusing on these controversies would shed light on the mechanism of depression among the older population.

In terms of demographic variables, our main finding is that geographical location and rural/urban community were the only relatively important predictor in the prediction of depression, which is a key point for our findings. Home-based older adults who live in western region were more vulnerable to depression compared to their peers in other regions. A possible reason might be that compared with those in the eastern and middle region in China, people in the west were living with less income, poorer health care support and worse living conditions [64], which would result in severer depressive condition. However, it is a pity to regard socioeconomic predictors, which showed less predictive values.

Our study could provide reference value for clinical practice. The identified risk factors can be used to inform the community prescribers, e.g., using self-reported measures along with inexpensive cognitive testing for episodic memory and mental intactness, and to target preventive interventions for improving the remission of depression. Self-reported information including sleep time, self-reported memory, CESD-10 score and geographical location. Specially, such identified risk factors and training model could be used for developing risk assessment tool (e.g., risk calculator or APP system), which likely including four modules of “individual information input”, “data management”, “disease prediction”, and “disease intervention”. Once the index data of elderly has been entered into the system, the individual development pattern of depression can transmit to community prescribers, which can help them complete the decision-making processes earlier, and adopt lifestyle intervention or clinical treatment for patients as soon as possible.

Most notably, this is the first study, to our knowledge, to investigate the hybrid ML model for depression estimation targeting a home-based population, particularly introducing a deep neural network algorithm (LSTM) to construct model based on 7-year longitudinal survey data in Chinese community-dwelling older adults. Furthermore, understanding the temporal dependence of theses predictors and what estimates a more worsening depression pattern is essential for designing customized interventions aimed to improve quality of life in these elderly people. Moreover, our hybrid model focused on the long-term course before onset of depressive, including the predictors of multiple time point, can also help the community medical providers to find people with positive symptoms as early as possible.

However, it is also important to bear in mind the limitations of the current study. Firstly, the external validation in an independent dataset was not conducted in the current analysis. Although within-sample cross-validation is known to be an almost unbiased method of population generalizability [65], it may not completely be suitable for the different characteristics of data from different samples. Validating our findings in large and independent data is the next important study. Secondly, due to limitation in data amount and follow-up waves, ML algorithms such as random forest and LSTM may not be able to represent their forecasting potential. Moreover, depression-related treatment information during the study was not collected, therefore, we did not know if the change of depression was affected by treatment. Thirdly, we converted the CESD-10 score into a binary outcome variables since our study was aimed to explore the future depression risk for those who were free of depression over the past three years, which would be better for primary screening in general population. The CESD-10 only evaluates the week before the assessment. Because of that, more detailed information to the courses and degree of the depression may be lost and even depression may not be accounted for, while for a more reliable determination of depression, more frequent evaluations and longer periods of follow-up are required. In our future research, we aim to verify our models in clinical scenarios with larger sample size, more waves of follow-up information, multi-task learning, and multimodal characteristics, to improve the accuracy of predicting model.

## Conclusion

In conclusion, this study developed a two-step hybrid model based “LSTM+ML” framework in depression estimation for home-based elderly. Utilizing the easily accessible sociodemographic and health predictors, the two-step hybrid machine learning prediction showed potential to distinguish no depression from those with depression. The decision support system based on the hybrid models may be valuable for community medical providers.

## Supplementary Information

**Additional file 1.**

## Data Availability

The datasets generated and analysed during the current study are available at Peking University Open Research Data Platform. We confirm that our Data Availability Statement complies with the Experts Data Policy. You could contact the corresponding author (fangya@xmu.edu.cn) if you want to request the data from this study.
