# Supplementary material for: A hybrid machine learning model of depression estimation in home-based older adults: a 7-year follow-up study
Source: BMC Psychiatry. 2022 Dec 21;22:816. doi: 10.1186/s12888-022-04439-4 (PMC9768728; doi:10.1186/s12888-022-04439-4)
Supplement: Supplementary file 1 — Additional file 1. [file 12888_2022_4439_MOESM1_ESM.docx]

**Supplementary Table 1. The summary of missing values of different predictors for each wave**

| **Predictors** | **2011** | **2013** | **2015** | **Total** |
| --- | --- | --- | --- | --- |
|  | **N (%)** | **N (%)** | **N (%)** | **N (%)** |
| **Demographic variables** |  |  |  |  |
| Age, year | 0 (0.00) | 0 (0.00) | 0 (0.00) | 0 (0.00) |
| Sex | 0 (0.00) | 0 (0.00) | 0 (0.00) | 0 (0.00) |
| Rural/Urban Community | 0 (0.00) | 0 (0.00) | 0 (0.00) | 0 (0.00) |
| Geographical location | 0 (0.00) | 0 (0.00) | 0 (0.00) | 0 (0.00) |
| Marital status | 0 (0.00) | 0 (0.00) | 0 (0.00) | 0 (0.00) |
| **Social economy variables** |  |  |  |  |
| Education level | 0 (0.00) | 0 (0.00) | 0 (0.00) | 0 (0.00) |
| Household per capita income, yuan | 42 (1.65) | 16 (0.63) | 9 (0.35) | 67 (0.88) |
| Household registration | 0 (0.00) | 0 (0.00) | 0 (0.00) | 0 (0.00) |
| Occupation status | 6 (0.24) | 7 (0.27) | 2 (0.08) | 15 (0.20) |
| Medical Insurance | 5 (0.20) | 73 (2.86) | 0 (0.00) | 78 (1.02) |
| **Life style & health status variables** |  |  |  |  |
| Life satisfaction | 193 (7.57) | 11 (0.43) | 4 (0.16) | 208 (2.72) |
| Self-reported health status | 1 (0.04) | 1 (0.04) | 0 (0.00) | 2 (0.03) |
| Social activities | 1 (0.04) | 0 (0.00) | 0 (0.00) | 1 (0.01) |
| Smoking | 0 (0.00) | 131 (5.14) | 2 (0.08) | 133 (1.74) |
| Drinking | 0 (0.00) | 0 (0.00) | 0 (0.00) | 0 (0.00) |
| Self-rated memory | 0 (0.00) | 4 (0.16) | 0 (0.00) | 4 (0.05) |
| Medical service | 29 (1.14) | 32 (1.26) | 25 (0.98) | 86 (1.13) |
| Sleeping time, hour | 7 (0.27) | 26 (1.02) | 13 (0.51) | 46 (0.60) |
| ADL disorder | 15 (0.59) | 0 (0.00) | 0 (0.00) | 15 (0.20) |
| Chronic disease | 0 (0.00) | 0 (0.00) | 0 (0.00) | 0 (0.00) |
| Disability | 2 (0.08) | 185 (7.26) | 205 (8.04) | 392 (5.13) |
| Cognitive ability | 0 (0.00) | 0 (0.00) | 0 (0.00) | 0 (0.00) |

**Supplementary Table 2. predictors in 2011 and univariate analysis of association with depression in 2018**

| Variables | Non-depression (n=1,655) | depression (n=893) | Total | Test of association |
| --- | --- | --- | --- | --- |
|  | **% or Mean(SD)** | **% or Mean(SD)** |  |  |
| Demographic variables |  |  |  |  |
| Age, year |  |  |  | χ^2^=0.742 |
| 60- | 80.72 | 81.97 | 81.16 |  |
| 70- | 18.25 | 16.91 | 17.78 |  |
| 80- | 1.03 | 1.12 | 1.06 |  |
| Sex |  |  |  | χ^2^=14.324*** |
| Male | 57.10 | 49.27 | 54.36 |  |
| Female | 42.90 | 50.73 | 45.64 |  |
| Rural/Urban Community |  |  |  | χ^2^=41.037*** |
| Rural | 58.61 | 71.44 | 63.11 |  |
| Urban | 41.39 | 28.56 | 36.89 |  |
| Geographical location |  |  |  | χ^2^=43.580*** |
| Eastern | 37.59 | 26.20 | 33.60 |  |
| Central | 34.62 | 35.28 | 34.85 |  |
| Western | 27.79 | 38.52 | 31.55 |  |
| Marital status |  |  |  | χ^2^=3.196 |
| Single | 15.71 | 18.48 | 16.68 |  |
| Married | 84.29 | 81.52 | 83.32 |  |
| Social economy variables |  |  |  |  |
| Education level |  |  |  | χ^2^=58.079*** |
| Low | 71.12 | 84.66 | 75.86 |  |
| High | 28.88 | 15.34 | 24.14 |  |
| Household per capita income, yuan |  |  |  | χ^2^=43.425*** |
| <5000 | 56.55 | 68.65 | 60.79 |  |
| 5000-10000 | 16.62 | 15.23 | 16.13 |  |
| >10000 | 26.83 | 16.12 | 23.08 |  |
| Household registration |  |  |  | χ^2^=41.330*** |
| Agriculture | 71.24 | 82.75 | 75.27 |  |
| Non-Agriculture | 28.76 | 17.25 | 24.73 |  |
| Occupation status |  |  |  | χ^2^=20.534*** |
| Agricultural work | 51.30 | 60.02 | 54.36 |  |
| Non-agricultural work | 10.88 | 7.28 | 9.61 |  |
| Retired | 34.92 | 3.57 | 33.40 |  |
| Unemployment | 2.90 | 2.13 | 2.63 |  |
| Medical Insurance |  |  |  | χ^2^=0.070 |
| No | 3.93 | 4.14 | 4.00 |  |
| Yes | 96.07 | 95.86 | 96.00 |  |
| Life style & health status variables |  |  |  |  |
| Life satisfaction |  |  |  | χ^2^=100.690*** |
| Satisfied | 8.16 | 21.61 | 12.87 |  |
| Medium | 64.53 | 59.46 | 62.76 |  |
| Not satisfied | 27.31 | 18.93 | 24.37 |  |
| Self-reported health status |  |  |  | χ^2^=155.614*** |
| Good | 27.43 | 12.32 | 22.14 |  |
| Fair | 52.93 | 47.37 | 50.98 |  |
| Poor | 19.64 | 40.31 | 26.88 |  |
| Social activities |  |  |  | χ^2^=16.087*** |
| Never | 50.03 | 58.34 | 52.94 |  |
| Ever | 49.97 | 41.66 | 47.06 |  |
| Smoking |  |  |  | χ^2^=5.307* |
| Never | 65.38 | 69.88 | 66.95 |  |
| Ever | 34.62 | 30.12 | 33.05 |  |
| Drinking |  |  |  | χ^2^=16.142*** |
| Never | 63.32 | 71.22 | 66.09 |  |
| Ever | 36.68 | 28.78 | 33.91 |  |
| Self-rated memory |  |  |  | χ^2^=72.291*** |
| Good | 29.00 | 45.91 | 34.93 |  |
| Fair | 48.64 | 39.98 | 45.60 |  |
| Poor | 22.36 | 14.11 | 19.47 |  |
| Medical service |  |  |  | χ^2^=17.041*** |
| No | 82.54 | 75.70 | 80.14 |  |
| Yes | 17.46 | 24.30 | 19.86 |  |
| Sleeping time, hour |  |  |  | χ^2^=83.410*** |
| 0- | 5.86 | 13.33 | 8.48 |  |
| 4- | 20.36 | 29.45 | 23.55 |  |
| 6- | 43.51 | 31.80 | 39.40 |  |
| 8- | 30.27 | 35.42 | 28.57 |  |
| ADL disorder |  |  |  | χ^2^=75.244*** |
| No | 86.34 | 72.34 | 81.44 |  |
| Yes | 13.66 | 27.66 | 18.56 |  |
| Chronic disease |  |  |  | χ^2^=30.054*** |
| No | 27.67 | 17.92 | 24.25 |  |
| Yes | 72.33 | 82.08 | 75.75 |  |
| Disability |  |  |  | χ^2^=9.663*** |
| No | 84.59 | 79.73 | 82.89 |  |
| Yes | 15.41 | 20.27 | 17.11 |  |
| Cognitive ability，score |  |  |  | F=49.804*** |
| Low | 43.81 | 58.45 | 48.94 |  |
| High | 56.19 | 41.55 | 51.06 |  |

**Notes.** χ^2^ denotes the Pearson’s Chi square statistic; **p* < 0.05, ***p* < 0.01, ****p* < 0.001.

**Supplementary Table 3. Model performance in predicting courses of depressive symptoms for sensitivity analysis on only participants with complete data**

| Model | Accuracy | Sensitivity | PPV | Brier | AUC (95% CI) |
| --- | --- | --- | --- | --- | --- |
| SVM | 0.721 | 0.489 | 0.466 | 0.188 | 0.705(0.702-0.706) |
| GDBT | 0.678 | 0.313 | 0.525 | 0.199 | 0.741(0.733-0.743) |
| RF | 0.755 | 0.222 | 0.670 | 0.167 | 0.751(0.747-0.753) |
| LR | 0.711 | 0.164 | 0.604 | 0.203 | 0.612(0.603-0.641) |

**Note.** RF: random forests, GBDT: gradient boosting decision tree, SVM: support vector machines, LR: logistic regression; PPV: Positive Predictive Value; CI: confidence interval; Parameter optimization: RF (ntree=200, mtry=20), SVM (C=0.5, γ=0.001), GBDT (α= 4, β=0.01, γ= 300).

**Supplementary Table 4. Details of predictor used in this study**

| **Variables** | **Abbreviation** | **Category** |
| --- | --- | --- |
| Age, years | AGE | 60-; 70- ; 80- |
| Sex | SEX | Male; Female |
| Rural/urban community | R/UC | Rural Community; Urban Community |
| Geographical location | GEO | Eastern; Central; Western |
| Marital status | MAR | Single (never married/divorced/separated and widowed); Married (married/partnered) |
| Education level | EDU | Low (elementary school and below); High (middle school and above) |
| Household per capita income, yuan | INC | 0-; 5000-; 10000- |
| Household registration | REG | Agriculture; Non-Agriculture |
| Occupation status | OCC | Agricultural work; Non-agriculturwork; Retired; Unemployed/Never work |
| Medical insurance | INS | Yes; No |
| Life satisfaction | SAT | Satisfied; Medium; Not satisfied |
| Self-reported health status | HEA | Good; Fair; Poor |
| Social activities | SOC | Never; Ever |
| Smoking | SMO | Never; Ever |
| Drinking | DRI | Never; Ever |
| Self-rated memory | MEM | Good; Fair; Poor |
| Medical service | SER | Yes; No |
| Sleeping time, hour | SLE | 0-; 4-; 6-; 8- |
| Activities of daily disorder | ADL | Yes; No |
| Chronic disease | CHR | Yes; No |
| Disability | DIA | Yes; No |
| Cognitive ability, score | COG | 0~21, high: score>10.5, low: score<10.5 |

**Supplementary Table 5. Observed indicators ^a^**

|  | Observed indicator |
| --- | --- |
| 1 | I was bothered by things that don’t usually bother me. |
| 2 | I had trouble keeping my mind on what I was doing. |
| 3 | I felt depressed. |
| 4 | I felt everything I did was an effort. |
| 5 | I felt hopeful about the future. |
| 6 | I felt fearful. |
| 7 | My sleep was restless. |
| 8 | I was happy. |
| 9 | I felt lonely. |
| 10 | I could not get “going”. |

**Note.** ^a^ Item 5 and item 8 had been reverse-scored before analysis.

**Supplementary Table 6. Optimal hyperparameters of the LSTM model**

| **Hyperparameter** | **Value** | **Remark** |
| --- | --- | --- |
| Learning rate | 0.0001 | Iteration steps |
| Batch size | 16 | Gradient descent method update weight w and deviation b |
| Training iterations | 78 | A total of 78 sets of data |
| Epoch | 100 | The total number of iterations is 78×100 |
| LSTM unit numbers | 20 | 20 data |
| LSTM unit dimensions | 40 | Independent variable dimension |

**Supplementary Table 7. The hyperparameter tuning strategy of three ML algorithms**

| - **SVM** | When fitting SVM model, hyper-parameters C and γ play substantial roles. To select the hyperparameters C and γ, we selected a sequence of values from 0.1 to 200 in intervals of 0.1 (C), and from 0.0001 to 0.1 in intervals of 0.0001 (γ) and tested all combinations of these values in cross-validation loop. The hyperparameters combination that maximized the AUC was selected for the final model. |
| --- | --- |
| - **RF** | The most important RF parameters, ie., the number of trees *n_tree_* and the number of variables to partition at each tree node *m_try_* were optimized to improve the performance. We selected a sequence of values from 1 to 500 in intervals of 1 ( *n_tree_*), and from 2 to 20 in intervals of 1 (*m_try_* ) and tested all combinations of these values in cross-validation loop. |
| - **GBDT** | During the establishment of GBDT, there are 3 main parameters that need to be tuned, such as learning_rate, n_estimators, and max_depth. α= max_depth, β=learning_rate, γ= n_estimators. α represents the maximum depth of each tree, and the nodes are divided when the depth of a single tree is less than α. β represents the contribution of each tree. γ represents the number of trees. The default parameter of the model learning rate is 0.1, in order to slow down the learning rate of the model and improve the accuracy of the model, the learning rate in this paper is set to 0.01. We selected a sequence of values from 1 to 500 in intervals of 1 ( γ ), and from 1 to 10 in intervals of 1 (α ) and tested all combinations of these values in cross-validation loop. |


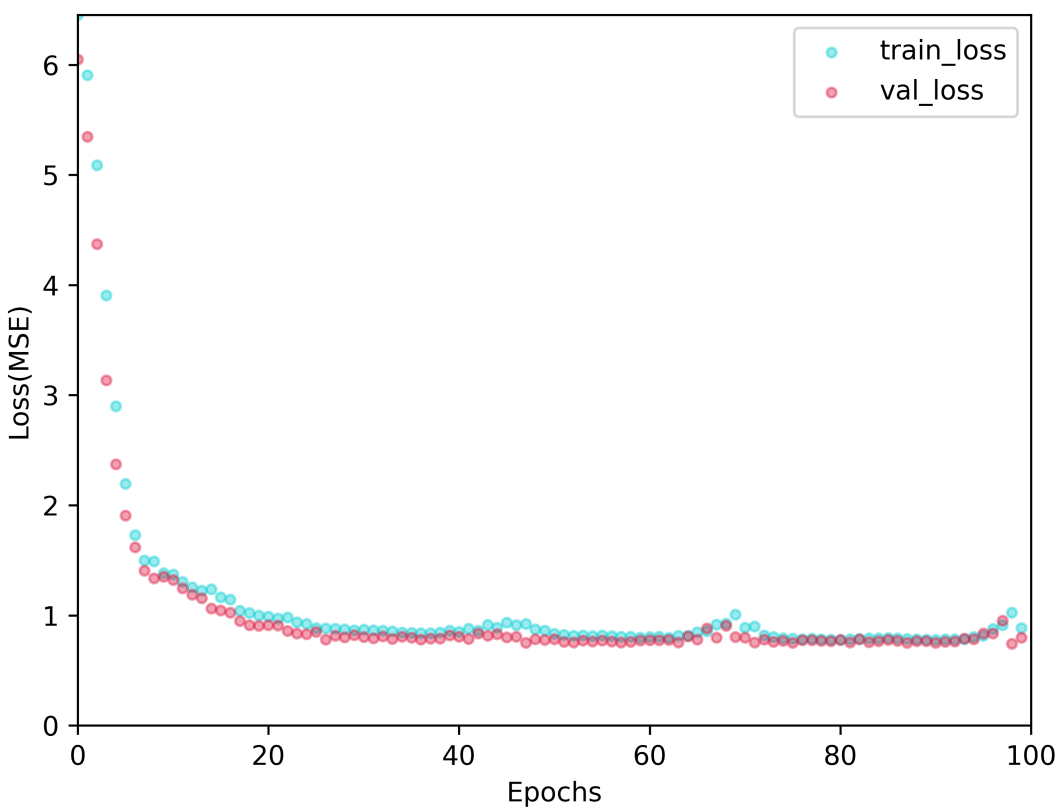


**Supplementary Figure 1.** The fitting curve of LSTM (MSE, Mean square error).


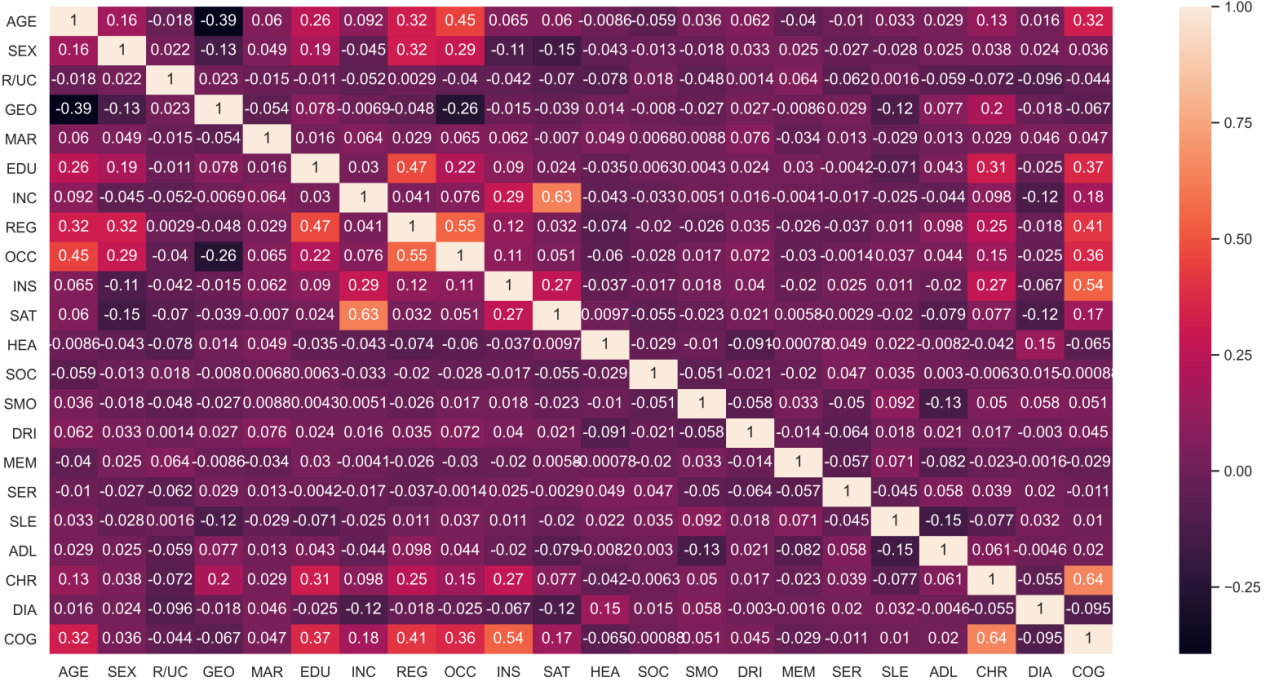


**Supplementary Figure 2.** The correlation coefficient matrix of each predicted risk factors by LSTM.
